# OnTrack Chile for people with early psychosis: a study protocol for a Hybrid Type 1 trial

**DOI:** 10.1186/s13063-022-06661-7

**Published:** 2022-09-05

**Authors:** Franco Mascayano, Iruma Bello, Howard Andrews, Diego Arancibia, Tamara Arratia, María Soledad Burrone, Sarah Conover, Kim Fader, Maria Jose Jorquera, Mauricio Gomez, Sergio Malverde, Gonzalo Martínez-Alés, Jorge Ramírez, Gabriel Reginatto, Alexandra Restrepo-Henao, Robert A. Rosencheck, Sara Schilling, Thomas E. Smith, Gonzalo Soto-Brandt, Eric Tapia, Tamara Tapia, Paola Velasco, Melanie M. Wall, Lawrence H. Yang, Leopoldo J. Cabassa, Ezra Susser, Lisa Dixon, Rubén Alvarado

**Affiliations:** 1 Department of Epidemiology, Mailman School of Public Health, Columbia University, New York, USA; 2 New York State Psychiatric Institute, New York, USA; 3 Columbia University Vagelos College of Physicians and Surgeons, New York, USA; 4 Department of Biostatistics, Mailman School of Public Health, Columbia University, New York, United States; 5 Instituto de Ciencias de la Salud, Universidad de O’Higgins, Rancagua, Chile; 6 Research and Postgraduate Institute, Faculty of Health Sciences, Universidad Central, Santiago, Chile; 7 Silberman School of Social Work, Hunter College, New York, USA; 8 School of Public Health, Faculty of Medicine, Universidad de Chile, Santiago, Chile; 9 Epidemiology Research Group, National School of Public Health, Universidad de Antioquia, Medellin, Colombia; 10 Research, Education and Clinical Center, VA New England Mental Illness, West Haven, CT USA; 11 Department of Psychiatry, Yale University School of Medicine, New Haven, CT USA; 12 School of Global Public Health, New York University, New York, USA; 13 George Warren Brown School of Social Work, Washington University in St. Louis, St. Louis, MO USA; 14 Department of Public Health, School of Medicine, Faculty of Medicine, Universidad de Valparaíso, Valparaíso, Chile

**Keywords:** Early psychosis, Hybrid Type 1 trial, Global mental health, Specialized coordinated services for first episode psychosis, Coordinated specialty care

## Abstract

**Background:**

Substantial data from high-income countries support early interventions in the form of evidence-based Coordinated Specialty Care (CSC) for people experiencing First Episode Psychosis (FEP) to ameliorate symptoms and minimize disability. Chile is unique among Latin American countries in providing universal access to FEP services through a national FEP policy that mandates the identification of FEP individuals in primary care and guarantees delivery of community-based FEP treatments within a public health care system. Nonetheless, previous research has documented that FEP services currently provided at mental health clinics do not provide evidence-based approaches. This proposal aims to address this shortfall by first adapting OnTrackNY (OTNY), a CSC program currently being implemented across the USA, into OnTrackChile (OTCH), and then examine its effectiveness and implementation in Chile.

**Methods:**

The Dynamic Adaptation Process will be used first to inform the adaptation and implementation of OTCH to the Chilean context. Then, a Hybrid Type 1 trial design will test its effectiveness and cost and evaluate its implementation using a cluster-randomized controlled trial (RCT) (*N* = 300 from 21 outpatient clinics). The OTCH program will be offered in half of these outpatient clinics to individuals ages 15-35. Usual care services will continue to be offered at the other clinics. Given the current COVID-19 pandemic, most research and intervention procedures will be conducted remotely. The study will engage participants over the course of 2 years, with assessments administered at enrollment, 12 months, and 24 months. Primary outcomes include implementation (fidelity, acceptability, and uptake) and service outcomes (person-centeredness, adherence, and retention). Secondary outcomes comprise participant-level outcomes such as symptoms, functioning, and recovery orientation. Over the course of the study, interviews and focus groups with stakeholders will be conducted to better understand the implementation of OTCH.

**Discussion:**

Findings from this study will help determine the feasibility, effectiveness, and cost for delivering CSC services in Chile. Lessons learned about facilitators and barriers related to the implementation of the model could help inform the approach needed for these services to be further expanded throughout Latin America.

**Trial registration:**

www.ClinicalTrials.govNCT04247711. Registered 30 January 2020.

**Trial status:**

The OTCH trial is currently recruiting participants. Recruitment started on March 1, 2021, and is expected to be completed by December 1, 2022. This is the first version of this protocol (5/12/2021).

## Background

Absent early intervention, schizophrenia spectrum disorders often become long-term disabling conditions with profound impacts on individuals, their families, and society at large [1]. Loss of income and costs of services compound these impacts. Substantial data support early interventions for people experiencing first episode psychosis (FEP) to ameliorate symptoms and minimize disability in the early phase of psychosis [2], and trials of FEP programs have been widely and successfully conducted in high-income countries (HICs) [3–5]. In Latin America, Chile is the only country that offers universal access to FEP services.

Chile has been one of the leaders of mental health reform in Latin America, serving as a model for the region [6]. Most of its population receive health and mental health care via the public system into which we plan to introduce evidence-based FEP services. Almost everyone living in the poorest or “marginal” communities in urban areas in Chile utilizes the public system. In terms of FEP, Chile has implemented two fundamental reforms that facilitate implementing and scaling up an evidence-based program. First, as of 2005, government legislation mandates that any person with FEP has the right to community-based treatment in the public health system free of charge or with an affordable co-payment [7] and requires that any person who is under evaluation for FEP be recorded in a “national FEP register.” This register records useful information, including the number of people with FEP identified annually in every locale of Chile [7]. Second, psychiatric hospital beds have been progressively replaced by an extensive network of outpatient mental health services in the public health care system [6] and psychiatric units within general hospitals. Mental health services are offered within a primary care system that covers the entire country. The primary care clinics are affiliated with specific mental health outpatient clinics to which they refer individuals they have identified as needing mental health services. This creates a single point of entry into mental health services for the population, including people with FEP. This centralized system offers an opportune environment for examining the implementation and scale-up of evidence-based FEP services in Chile. Furthermore, it will inform how to build structures for FEP services in other Latin American countries, many of which are already moving in this direction even those with less resources.

Nonetheless, previous studies have documented two crucial gaps in FEP services in Chile. First, primary care providers are not adequately trained to identify and refer individuals with FEP to outpatient clinics [8]. Second, the mental health services being provided for people with FEP are not up to date and not totally supported by current evidence [9]. For example, although over 80% of people treated for FEP in Chile receive medications, only 40% receive other important services such as support for education and employment, family counseling, and peer support [8, 9]. Moreover, when these other services are offered, they tend to be ad-hoc because most mental health providers are not appropriately trained in evidence-based approaches (i.e., psychosocial interventions) for psychosis [9, 10]. This is the gap that is being addressed by the current study by first adapting OnTrackChile (OTCH) from OnTrackNY (OTNY), a coordinated specialty care (CSC) program for FEP currently being implemented across the US, and then examining its effectiveness and implementation across five different regions in Chile. OTNY is appropriate for adaptation to OTCH because it is (1) an evidence-based program with high rates of engagement among users associated with improvements in symptoms and functional outcomes [2, 11]; (2) scaled up to serve individuals throughout New York state by working closely with state and local mental health authorities [12]; and (3) practical and scalable with good fidelity [13].

Accordingly, the overarching goal of this Hybrid Type 1 trial is to evaluate the effectiveness and implementation of OTCH in 21 mental health clinics. We will use the Dynamic Adaptation Process model [14] to inform the adaptation and implementation of OTCH in the Chilean context, and then use a cluster-RCT (*N* = 300) to test the model’s effectiveness and cost. The primary outcomes in the cluster-RCT will be implementation outcomes (fidelity, acceptability, and uptake) at the provider- and participant-level, and service outcomes (patient-centeredness, adherence, and retention) at the participant-level. We will also examine moderators of implementation at the community-, provider-, and participant-level. Secondary outcomes will be clinical effectiveness including symptoms, functioning, and recovery orientation at the participant-level. We will also test whether implementation and service outcomes are mediators of clinical effectiveness and estimate the total and incremental costs of the intervention. Finally, we will use a set of qualitative measures to learn about the experiences with FEP services among clients, family members, providers, and policy makers.

## Research objectives

Aim 1: Evaluate the implementation of OTCH at 12 months and 24 months in a cluster-RCT.

- Compare OTCH vs Usual FEP care on service and implementation outcomes.
- Examine whether factors at community-, provider-, and participant-level moderate service and implementation outcomes of OTCH vs Usual FEP care.

Aim 2: Evaluate the effectiveness of OTCH vs Usual FEP Care at 12 months and 24 months.

- Compare the effectiveness of OTCH vs Usual FEP care on symptoms, functioning, and recovery orientation.
- Test both Service and Implementation Outcomes at 12 months as mediators of the effectiveness of OTCH vs. Usual FEP care at 24 months.

Aim 3: Estimate the cost of delivering OTCH vs Usual FEP care over a 24-month period.

## Methods/design

### Study design

This effectiveness-implementation Hybrid Type 1 trial aims to examine both the implementation and effectiveness of OTCH. A Hybrid Type 1 design, as we propose, tests the effects of a clinical intervention on client-level outcomes, while at the same time exploring multi-level implementation factors that can inform and promote the use of the intervention in real-world settings. It has been proposed as a solution to accelerate the process of transferring evidence-based practices into usual care. Moreover, the Dynamic Adaptation Process (DAP) [14] will be used to adapt and implement OTCH, as well as to examine fidelity and document implementation barriers and facilitators. The DAP provides a way to thoroughly identify and incorporate adaptations at multiple levels and facilitate implementation across the whole study. In contrast to most dissemination and implementation models, within the DAP, modifications, and adaptations are made by a team exclusively devoted to this task known as “Research Adaptation Team,” who is composed of multiple stakeholders and aimed to reflect what was learned about: a) understanding contextual conditions, and how context might be modified; and b) how these conditions might modify the nature of the content of the intervention curriculum.

**Fig. 1:**
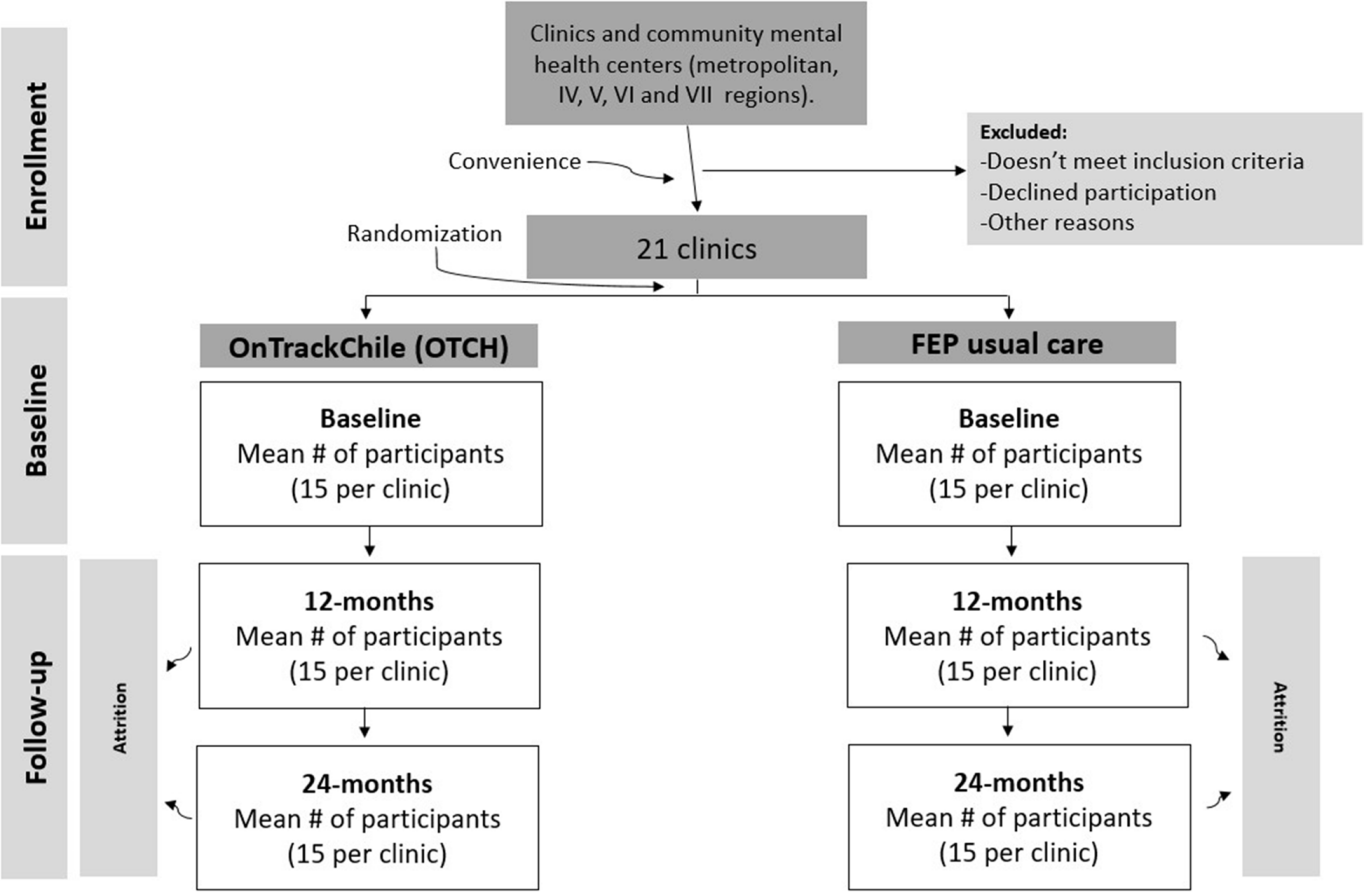
Flow diagram of the cluster RCT

A cluster-RCT will be conducted among 300 participants with FEP drawn from 21 outpatient clinics that include marginalized and poor communities to test the effectiveness of OTCH (see Fig. 1 below based on PRISMA guidelines).

### Description of sites

Twenty-one outpatient clinics located in five different regions in Chile will be enlisted in this study. The regions involved in the study include the Metropolitan Region of Santiago with a population of 7,112,808; the IV region of Coquimbo with a population of 757,586; the V region of Valparaíso with a population of 1,815,902; the VI region of O’Higgins with a population of 914,555; and the VII region of Maule with a population of 1,044,950. In these regions, more than 80% of the total population uses the public health system. Thirteen of the 21 clinics are in the Santiago Metropolitan region, which is divided in 4 health districts: Southern, Western, Southeastern, and Central Metropolitan. Further information regarding population served, poverty level, and expected enrollment for each of the twenty-one clinics is available upon request.

### Adaptation process

**Fig. 2:**
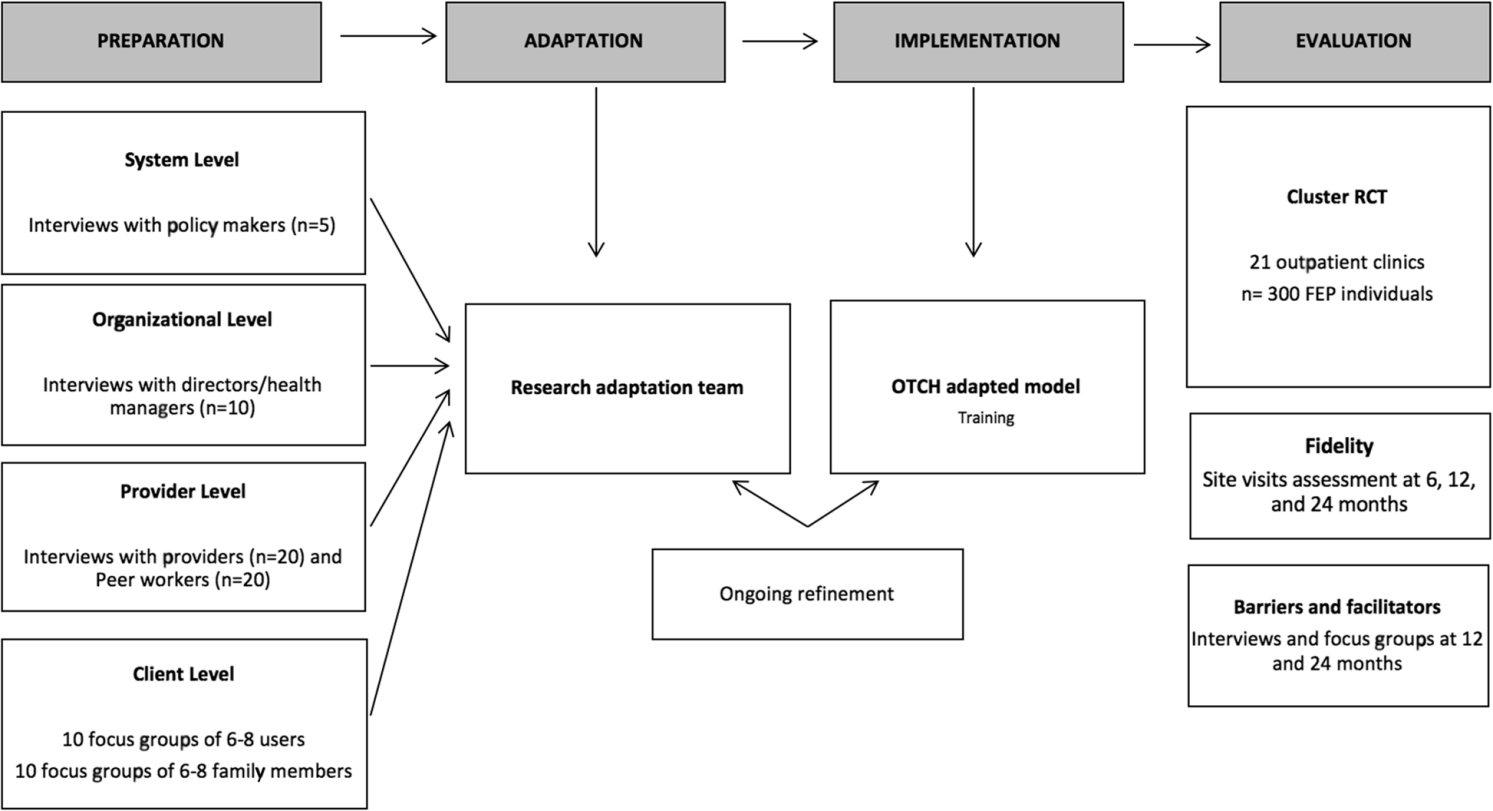
Dynamic Adaptation Process

As noted previously, we will use the Dynamic Adaptation Process (DAP) to adapt and implement OTCH, examine fidelity, and document barriers and facilitators. The implementation of OTCH will include four phases: Preparation, Adaptation, Implementation, and Evaluation (Fig. 2). Ongoing experience will iteratively inform adaptation as needed. We will convene multiple stakeholders (e.g., policy makers, providers, clients) in qualitative interviews and focus groups to discuss contextual conditions and identify potential modifications to the OTCH model and training curriculum. Then, once OTCH teams are established, a centralized training team will provide ongoing training using a virtual learning platform which will facilitate synchronous and asynchronous learning opportunities including virtual meetings, treatment manuals, tools, videos and other resources to support implementation. We will use mixed methods to examine to examine the implementation of OTHC to identify facilitators and barriers throughout the trial and to capture adaptation through the trials. In line with the Dynamic Adaptation Process, the qualitative data collected during the preparation phase will be presented and discussed with the OTCH clinical team as they are establishing their implementation to determine areas for adaptation via consensus. As the teams are implementing the OTCH model, the research team will use the DAP to allow us to systematically evaluate modifications made and ascertain their impact. Our approach captures processes for modification including reasons for the modification, what was modified, level of modification, and timing across phases of treatment. We also continue to examine the implementation and adaptations carried forth through OTCH teams during the 6 and 18-month qualitative data collection activities.

### Sample size and power analysis

The first phase of the study which focused on the qualitative data collection did not include any formal power calculation to determine the sample size. Instead, we took a multistakeholder approach to include participants at different levels involved in the care for people with FEP including users and their family members. To incorporate a variety of stakeholder perspectives, we aimed to conduct 10 focus groups with 6–8 stakeholders.

For the cluster trial, power was calculated conservatively based on the participation of 270 participants (rather than the full 300 to be enrolled), anticipating a possible participant drop-out of ~10%. We estimated that, with at least 10 clinics in each condition, we will have 80% power to detect a difference in mean outcomes between study conditions with a moderate effect size of Cohen’s *d* = 0.35 following the standard detectible effect size formula for clustered randomized trials [15, 16]. This power calculation was derived assuming an intra-class correlation of 0.05 for outcomes by clinic (15 participants nested within 21 clinics). We also assume an alpha of 0.05 and that all tests are 2-tailed. Previous research has shown interventions like OTCH to have moderate to large effects on implementation and service outcomes, such as medication adherence, service utilization, and engagement in treatment [5, 17]. We have also shown that FEP providers in Chile rely primarily upon medication and are usually not trained to promote shared decision-making, recovery orientation, or person-centered care [9]. Then it is reasonable to anticipate at least moderate (and probably large) effects of OTCH on these indicators of implementation. Finally, evidence for the expected effect on participant-level symptoms and functioning comes from the RAISE Connection Program study, which indicated improvement over 24 months for participants enrolled (e.g., PANSS improvement effect size 0.91, occupational functioning 1.2, and social functioning 0.72) [17]. Power to detect mediation effects (Aim 2b) depends on the size of the effect of intervention on implementation and service outcomes (path a), and the correlation between implementation and services outcomes and participant level symptoms, functioning, and recovery (path b). Based on tabulated recommendations for sample sizes needed to test mediation effects with a sample of size *N*=270 with follow-up data, we have over 80% power to detect a significant mediation effect with path a=.26 and b=.26. Path a is expected to be >0.35 (argued for Aim 1 above), and we expect based on our previous work that there will be at least moderate associations between implementation and clinical outcomes (*r*>0.40), assuming no confounding of mediator-outcome relationship.

### Randomization

The 21 participating clinics will first be divided into two groups based on the poverty level of the catchment area and expected enrollment of individuals with FEP for each clinic. The largest site will be matched with two clinics of a similar poverty level and clinic size as well, as noted above. The matching will be done based on a visual inspection of a scatter plot of expected enrollment by the income of each site. Then, blocks of size 2, and one block of size 3, will be randomly generated. Randomization will be performed using a randomization scheme generated by SAS 9.4. This procedure will entail (1) generating a random number 0 or 1 ten times, and (2) assigning 0 to the Usual FEP care clinic and 1 to the OTCH clinic in each matched pair. For the block of size 3, two sites will be assigned to either OTCH or Usual FEP care, and the remaining site to either OTCH or Usual FEP care as well. The statistician will notify the contact-PI once the randomization is done, and he will then call the CMHC Director to inform him/her of the assigned treatment status, followed by a written note to the Director with the same information. The random allocation within blocks of two will enable us to bring into the study one OTCH and one Usual FEP Care CMHC per month. We note that the randomization (and notification) will be done at the start of the project, which will enable us to select equal numbers of OTCH and Usual FEP Care CMHCs for data collection (at organizational, provider, and participant level) during the preparatory phase.

### Inclusion/exclusion criteria

Study inclusion criteria for clients are (1) verified as having FEP by a psychiatrist in a mental health clinic within 2 years of the onset of psychosis; (2) meet ICD-10 criteria for a diagnosis of schizophrenia, schizophreniform, schizoaffective disorder, delusional disorder, brief psychotic disorder, or psychosis not otherwise specified; (3) 15–35 years old; (4) have the capacity to provide fully informed consent (for those under 18, assent plus informed consent of parent/guardian); and (5) able to participate in research assessments in Spanish. In order to conform with government legislation, individuals will be deemed ineligible if they have a non-psychiatric medical condition that impairs functioning, a psychosis due solely to another medical condition, or a developmental disability. All subjects aged 18 years or older will be required to provide written informed consent for study participation. Subjects under 18 will provide written assent and their parent/guardian written consent. As noted above, we will also enroll providers, who will be mental health workers who have direct contact with FEP clients. Of note, these two groups (clients and providers) will be recruited at both OTCH and Usual FEP care sites to assess the implementation outcomes included in this cluster RCT (i.e., fidelity, acceptability, and uptake).

For the qualitative assessments, we will interview clients enrolled in the cluster RCT, plus policy makers and health directors. These will be individuals who are 18 years of age or older and employed full- or part-time at public agencies or participating clinics, respectively. Finally, we will also interview family members, who will be individuals who live with or are close to the client, and who are involved in providing support.

### Interventions: OTCH vs usual FEP care

#### OTCH

We will adapt the OTNY model to the Chilean cultural and service context to create OTCH. OTNY, as previously noted, is an intensive outpatient program for young people who have experienced their first episode of psychosis. The program is implemented by a multidisciplinary team with specialized training, who provide coordinated evidence-based treatments based on the interests, needs, and preferences of each participant. The program emphasizes assertive outreach and engagement and is offered for an average of 2 years.

OTNY teams offer participants a range of evidence-based treatment options, including individual therapy based on Cognitive Behavioral Therapy (CBT) for psychosis, psychiatric medications, family education, and support, supported education and employment, case management and community support, and a focus on physical wellness and coordination with primary health. OTCH teams will be able to offer all of these services, some of them with low availability in the country (e.g., CBT), except peer support since clinics are not staffed with peer specialists. Services are delivered in a person-centered, recovery oriented, culturally competent manner that prioritizes an individual’s needs and treatment preferences using shared decision-making [13]. The overarching goal is to help participants achieve personalized goals typically related to school, work, and relationships. The program strives for a balance between encouraging independence and offering support when needed [13]. An OTCH team will be comprised of five members however, adaptations will be made to how the roles will be carried out by the respective team members in response to the local context (Table 1).

**Table 1:** OTCH team roles

Role	Description
Team coordinator (TC)	A mental health professional, selected by a health director/manager, who will oversee the OTCH team and ensure that all team members adequately fulfill their roles.
Primary clinician (PC)	Preferably a psychologist, who will be the primary resource for the participant and his/her family and responsible for formulating the treatment plan in accordance with the participant’s preferences and coordinate the execution of the treatment plan with the rest of the team. This professional will also deliver psychosocial interventions to participants and family education and support. Some teams may have more than one professional fill this role.
Community support professional (CSP)	Preferably an occupational therapist, who will provide supported employment and/or education and connect the participant with peers and community resources. In the OTNY model, this role is known as the “supported employment and education specialist.” The name change for OTCH reflects an expansion of this role in the Chilean adaptation.
Prescriber	A psychiatrist, who will provide medications and symptoms management using shared decision-making and an evidence-based prescribing approach.
Nurse	Either carried out by a nurse or a nursing technician, who will support the psychiatrist and monitor medication use and side effects, facilitate individual and group wellness sessions, monitor physical health and vital signs, and coordinate with primary care.

Delivery of OTCH services will be done using a phased approach; for details on each phase see Table 1. At the beginning of treatment, a participant will be assigned a Primary Clinician (PC) by the Team Coordinator (TC) and meet with the PC, and ideally the Prescriber, for an initial orientation session. During this initial phase, team members focus on engaging with participants and families, assessing their needs, and helping the individual begin identifying goals. Because no part of the model is compulsory, program participants will be encouraged to use shared decision-making to identify treatment areas to prioritize. Even if a participant decides not to take medication, he/she will still be encouraged to meet with the Prescriber and Nurse even if infrequently. For instance, participants should meet with the PC and Prescriber/Nurse once a week for the first month (the prescriber and nurse will alternate weeks) and then every 2 weeks thereafter. Meetings with the Community Support Professional (CSP) will depend on each participant’s goals, interests, and preferences. This person-centered, recovery-oriented approach is crucial to foster engagement with services within and outside OnTrack.

The second phase constitutes the bulk of the 2-year program. Once a strong relationship has been established with the participant and family and individualized goals have been defined, team members will provide interventions focused on helping individuals achieve their goals and promote their recovery as well as deliver core sessions providing psychoeducation about FEP, its course, treatment and information to support recovery and resilience to participants and family members. During this time period, meetings with the PC, Prescriber/Nurse, and other team members will continue to happen as frequently as needed depending on the interventions being delivered and participant/family preferences. As participants start reaching goals and becoming more involved in community and school/work activities it is typical that meetings with team members decrease in frequency. The final phase begins six months prior to discharge from the program and is focused on understanding the participant’s achievements, and the identification of future needs to determine connection to long-term services.

The intervention is offered in a flexible manner, responsive to the needs and preferences of each participant which helps to limit disengagement from services. If participants decide that they do not want to take engage in any part of the model, the services offered will be modified to meet this request. Participants should not receive other concomitant psychiatric treatment during the time that they are receiving care from the OTCH team because the interventions provided by the team are comprehensive and should treat primary psychosis and other psychiatric comorbidities. Criteria for discontinuation of the intervention would be based on clinical judgment indicating that (1) the diagnostic presentation has changed, and the individual has a different psychiatric disorder requiring different services or (2) the worsening of symptoms requiring a different level of care such as inpatient hospitalization. Furthermore, if a participant decides that they no longer want to receive services from the OTCH team, an appropriate referral and linkage will be made either to another service within the community mental health center or to another mental health service provider (Table 2).

**Table 2:** Typical activities of each phase of the intervention

Phase 1: Engagement and needs assessment (Months: 1–3)	Phase 2: Ongoing intervention and monitoring (Months: 3–18)	Phase 3: Transition (Months: 18–24)
Activities: • Introduction to all team members • Description of OTCH program • Intake assessment • Needs assessment (e.g., housing, income, etc.) • Risk and trauma assessment • Building rapport and getting to know the participant and their support network • Identification of individual goals (e.g., employment, education, relationships) • Introduction of shared decision making • Deciding on level and type of family involvement • Development of a treatment plan	Activities: • Delivery of core psychoeducation sessions • Delivery of psychosocial interventions to help achieve goals and build resiliency (e.g., social skills training, CBTp, substance use treatment, illness self-management, coping skills, and behavioral activation) • Suicide prevention • Review and revise treatment plan • Actively pursue work and school goals • Delivery of psychoeducation and support to family • Relapse prevention • Explore transition readiness	Activities: • Determine gains made and goals attained • Decrease frequency of services delivered • Develop a transition plan • Identify and connect to mental health services in the community • Identify and connect to community-based supports • Engage family in transition plan and identify their role • If possible, test whether these new services align with participant’s preferences

### Training and supervision

The training approach for the OTCH teams will follow the established training strategies utilized by the OnTrack Central training team at the Center for Practice Innovations, for training teams in the state of New York and nationally [18]. The training will be delivered primarily through an online learning platform that will allow for virtual meetings focused on individualized and group consultation and coaching, didactic webinars, opportunities for sharing and learning from each other, and discussing data to help maximize the quality of the implementation. The OTCH trainers will also be available via email or phone for specific consultation needs that arise over the duration of the program and they will have access and regular meetings with OnTrack Central trainers in New York to help adapt the training strategy to meet the local needs.

OTCH teams will receive an initial, remote training providing a general overview of the treatment model before starting implementation. This allows for an opportunity to discuss practical components of the implementation, understand where each team is at regarding their readiness for implementation of the treatment model and provides an opportunity for engaging in in vivo problem solving to address questions and barriers faced at the individual agencies hosting the programs. Training then continues following a set monthly schedule of role and team-based learning collaborative calls, so that each team receives the same amount of training and information on a regular basis (Table 3). Learning collaboratives have traditionally been used in health care and are increasingly used in behavioral health care [18]. Fundamental elements of a learning collaborative include a number of organizations working together, using quality improvement methods to close the gap between potential and actual performance, learning from experts as well as from one another, and using data to track performance [18]. This will be complemented with resources for self-paced learning that teams can access through the virtual learning platform. Trainers and trainees will also receive site-level data (both related to outcomes and fidelity) that will be utilized during virtual meetings to guide discussions focused on improving the implementation, clarifying questions related to the model, and problem-solve challenges they may be facing.

**Table 3:** Description of training approach

Training event	Description
Initial Implementation Calls	Consists of individual phone calls which provide an opportunity for the agency leadership and the team leader to develop a strategy for forming the team and identifying the agency-level infrastructure that needs to be in place to ensure good team functioning.
Synchronous and Asynchronous Initial Training	Consists of a 3-month training period where individuals will be assigned materials for self-paced learning and then will join a monthly videoconference meeting. Training will consist of a general overview of the treatment model and all of its components delivered before starting implementation of OTCH.
Individual role-based videoconference meetings	Consists of monthly learning collaborative virtual meetings facilitated by a trainer focused on the implementation of specific elements of the role and give providers an opportunity to learn from each other’s experiences
Care consultation videoconference meetings	Consists of monthly virtual meetings attended by two entire teams and at least two trainers focused on discussing a program participant in detail and getting advice and feedback
Special topic webinars	All teams are invited to receive training on a specific topic requested by trainees (e.g., prescribing long-acting injectable medications, cultural competency, and suicidality). These will take place as needed.

#### Usual care

The FEP policy will ensure that participants from the control sites will have access to Usual FEP Care. This is generally provided in mental health outpatient clinics, which (i) serve a population enrolled in the public health care system, (ii) provide services such as psychiatric medication, psychotherapy, and psycho-education for people with FEP and their families, and (iii) have a diverse team of professionals that include psychiatrists, psychologists, psychiatric nurses, social workers, occupational therapists, and family counselors. The services offered in these clinics, however, tend to have some limitations, three of which are relevant to this trial.

First, these clinics offer services to clients with a wide range of mental health conditions, not just those with FEP, and therefore, providers lack specialized training on treating FEP (e.g., evidence-based prescribing of antipsychotic medications or evidence-based psychosocial approaches). Second, recovery-oriented services based on shared-decision making and person-centeredness are rarely offered in these clinics since a more biomedical model of care tends to be used. Third, they tend to have minimal resources to provide services in the community and often face several challenges in delivering coordinated care between different types of services (i.e., primary care, other specialized mental health services, and emergency services) and likely across professionals in a team-based model.

### Recruitment

Given the current COVID-19 pandemic, most research and intervention procedures will be conducted remotely, but there might be some exceptions as described below. This was decided after consulting with local stakeholders, including providers from the participating clinics, who noted that many clinics are providing mental telehealth services via telephone, email, and video conferencing. Considering that most people in Chile have smart phones, this will be the method of choice to contact, interview, and follow up potential participants via either telephone or Zoom/Webex calls.

After a site enters the study, the participating clinic will be asked to refer all individuals with FEP to our study team. A health provider in each of the participating clinics will be designated to be the local point person for the coordination of recruitment and follow-up of study participants in his/her site; this role will be referred to as “Local Coordinator.” As noted previously, an FEP registry is in place in Chile to identify individuals with an FEP to ensure they receive timely and appropriate treatment. The Local Coordinators can use that database to identify potential participants at their clinics. Using this tool, the Local Coordinator will assign a numeric screening ID number to each potential participant and maintain a log with this information in a secure cabinet in each site. Each site will be assigned a numeric range (1000s, 2000s, 3000s, etc.). The Local Coordinator, who is not part of the OTCH research team, will contact the potential participants by phone, videoconference, or in-person when possible. If verbal consent is obtained, the Local Coordinator proceeds with a brief introduction to study. The Local Coordinator will briefly explain that he or she can participate in a research study and will describe the study’s purpose. If the client accepts to participate, the Local Coordinator will notify a member of the Research Team, known as the Registration Designee, who will oversee assessing the capacity of consent and administering the informed consent form. If someone serves as a Registration Designee for the participant, this person will not be allowed to serve as an interviewer for this participant in order to maintain the blind. This process will take place remotely unless the clinic is open for in-person appointments. Once consent is obtained, an interviewer will be notified so he/she can contact the potential participant and conduct the baseline assessment. Similar procedures will be followed for minors, and for providers, policy makers, health directors, and family members who participate in the study.

Following the recommendations by McRae et al. [17] and Giraudeau et al. [18] on administering consent forms in cluster RCTs, there will be two different consent forms: one for participants from intervention clinics and another one for participants from controls clinics. In addition, we will have other consent forms for policy makers, health directors, providers, and family members.

Interviewers who conduct both the clinical assessments and qualitative interviews will be mental health professionals with prior research training and experience conducting clinical assessments. They will be trained on research and assessment procedures by the Research Team through 3 on-line, videoconferencing sessions. The first session will entail going over the research protocol and the interviewer’s manual. The second session will address the required procedures to enter data into RedCap and report adverse events. Finally, in the third session, a thorough review of the quantitative and qualitative measures will take place, with special emphasis in the administration of the clinical-based measures such as the PANSS.

The risks to participants are minimal, and the measures we will take, combined with rigorous team training, will reduce them even further. In case of emotional upset and/or suicide feelings, immediate action will be taken, such as a referral for mental health and suicide risk assessment and appropriate counseling at usual health services in Chile.

### Data collection

#### For the trial

Table 4 provides a description of all the measures that will be used in the trial. All instruments will be administered at baseline 12 and 24 months. Selection of measures was guided by the PhenX toolkit for clinical measures in early psychosis and key moderators that are known to influence the implementation of evidence-based practices [19]. All measures will be translated into Spanish if a Spanish language version is not available using the WHO guidelines for translating and adapting measures.

**Table 4:** Study assessments

	Construct	Group	Measure description	When	Time
Implementation outcomes	Fidelity	Providers, families, and participants	Adapted version of the OTNY fidelity scale. The scale assesses the degree to which FEP services deliver evidence-based practices. Fidelity assessment will be based on providers’ feedback, supervision calls, and site visits. Site visits have been established as a best practice for fidelity assessment [20].	6, 12, 24 months	NA
	Acceptability	Providers	Providers’ attitudes to evidence-based practices will be measured by the *Evidence-Based Practice Attitude Scale* (EBPAS) [21]. The EBPAS consists of 15 items rated on a five-point scale from 0 (“Not at all”) to 4 (“Very great extent”). Range: 0–60 points.	Baseline, 12, 24 months	15 min
	Uptake	Participants	The *CollaboRATE* [22] is a 3-item scale that measures Shared-Decision Making from users’ perspective. Each of the three items is rated on a scale of 0 (“no effort was made”) to 9 (“every effort was made”). Range: 0–27 points.	Baseline, 12, 24 months	5 min
		Providers	The *Shared Decision Making Questionnaire – Physician Version (SDM-Q-Doc)* [23] will be employed to measure SDM at the provider level. This asks the clinician first to enter the health problem the consultation was about and which decision was made. The questionnaire continues with nine items about the steps in the SDM process, scoring at a six point scale that ranges from 0 (completely disagree) to 5 (completely agree). A total score can be calculated by summing the scores of all items. A high score indicates more SDM.	Baseline, 12, 24 months	10 min
		Providers and participants	The *Recovery Self-Assessment (RSA)* [24] provider and patient versions is a 32-item, self-administered rating scale that focuses on perceptions of recovery principles and overall quality of services. It captures both providers’ and patients’ perceptions that a specific recovery-oriented intervention, in this case OTCH, is agreeable, palatable or satisfactory. Each item is rated on a 5-point scale (1 = Strongly Disagree; 5 = Strongly agree). Higher scores indicating greater quality care. Range: 32–160 points.	Baseline, 12, 24 months	15 min
		Providers and partcipants	Ten ad-hoc questions regarding receipt of services that are central to OTCH including supported employment-education, family intervention/support, psychosocial interventions, and personal strenghts and resiliency training	Baseline, 12, 24 months	3 min
Service outcomes	Patient-centeredness	Participants	The *Youth Services Survey (YSS)* [25] is a scale that assesses perceptions about accessibility, quality, and impact of mental health service over a period of time. Only the first 15 items will be used in this study. Each item is rated on a 5-point scale from 1= “Strongly disagree” to 5 = “Strongly agree” (Range: 15–75 points).	Baseline, 12, 24 months	10 min
	Medication adherence	Participants	Measured by the *Brief Adherence Rating Scale (BARS)* [26] It consists of 4 items: 3 questions and an overall visual analog rating scale to assess the percentage of antipsychotic medication doses taken by the user in the past month (0–100%).	Baseline, 12, 24 months	5 min
	Retention	Participants	Time remaining in treatment will be estimated by counting the number of days between randomization to the time of the last mental health service received as defined by the RAISE-ETP report. The *Service Utilization and Resources Form for Schizophrenia (SURF)* [27] will be used to record users’ utilization of psychiatric and psychosocial rehabilitation services by conducting telephone contacts on a three-monthly basis. Range: from 0 to 365 days over a 12-month period; from 0 to 730 days over a 24-month period.	Every 3 months	15 min
Clinical outcomes	Psychotic symptoms	Participants	*Positive and Negative Syndrome Scale (PANSS)* [28] scale will be used to assess positive and negative symptoms (only the first 14 items). This scale includes seven rating points that represent increasing levels of psychopathology, from 1 (“absent”) to 7 (“extreme”). Range *7–*49 points.	Baseline, 12, 24 months	20 min
	Functioning	Participants	The *Social and Role Functioning in Psychosis and Schizophrenia* (SRFP) [29] scale asks for social, behavioral, and occupational difficulties associated with mental illness, including psychosis and schizophrenia. The scale rates functioning on a scale of 0 (“extreme role dysfunction”) to 10 (“superior social/interpersonal functioning”). Range: 7–70 points.	Baseline, 12, 24 months	20 min
	Recovery orientation	Participants	The *Questionnaire about the Process of Recovery (QPR)* [30] assesses users’ perceptions about recovery from psychosis. It has 15 items each scored on a 4-point scale ranging from 0 (“strongly disagree”) to 4 (“strongly agree”). Higher scores on the measure are indicative of recovery (Range: 0–60 points).	Baseline, 12, 24 months	15 min
Moderators	Poverty	Community-level	Administrative data from the *Chilean System of Social Protection* [31] will be utilized to estimate percentage of the population living below the national poverty lines (0–100%).	As available across study period	N/A
	Providers’ Attitudes to EBP	Providers	This will be measured by the *Evidence-Based Practice Attitude Scale* (EBPAS) [21] as described above under implementation outcomes.	Baseline	10 min
	Providers’ Recovery orientation	Providers	The *Recovery Self-Assessment (RSA)* [24] as described above under implementation outcomes	Baseline	15 min
	Symptoms	Participants	Measured by PANSS [28] as described above under clinical outcomes	Baseline	20 min
	DUP	Participants	The time between the onset of psychotic symptoms and initiation of treatment at a mental health clinic based on the FEP registry.	Baseline	NA
	Functioning	Participants	Measured by *SRFP* [29] as described above under clinical outcomes	Baseline	20 min
	Socio-demographics	Participants	Age (15–19, 20–24, 25–29, 30–34, 35–40 years), gender (male, female), ethnicity, education, employment, and marital status	Baseline	10 min

For estimating total health care costs, we will rely on both administrative data on expenditures for implementing and delivering OTCH, and interview data, collected every three months from participants on their use of services other than OTCH. Research assistants will document service use using the Service Use and Resource Form (Table 4) every three months, during the first year, to assure adequate recall [27, 32–39].

#### For the qualitative assessments

We will use qualitative methods to identify and characterize potential barriers/facilitators and processes that may inform the implementation of OTCH and its continuous adaptation process. In particular, we will explore factors at multiple levels (system-, organizational-, provider- and client/family member-level) by conducting semi-structured, on-line qualitative interviews with policy makers, director/health managers, and providers and focus groups or qualitative interviews with users and family members.

Qualitative interviews and focus groups will be conducted when OTCH teams begin enrollment activities and at 6- and 18-month post-randomization at all OTCH sites and at five sites randomized to usual care. We will conduct these interviews and focus groups via telephone/teleconferences. All interviews and focus groups will be audio recorded with participant’s informed consent. We will employ a purposive sampling approach to identify and invite key informants representing each group of participants [16–18].

### Data analysis

**Fig. 3:**
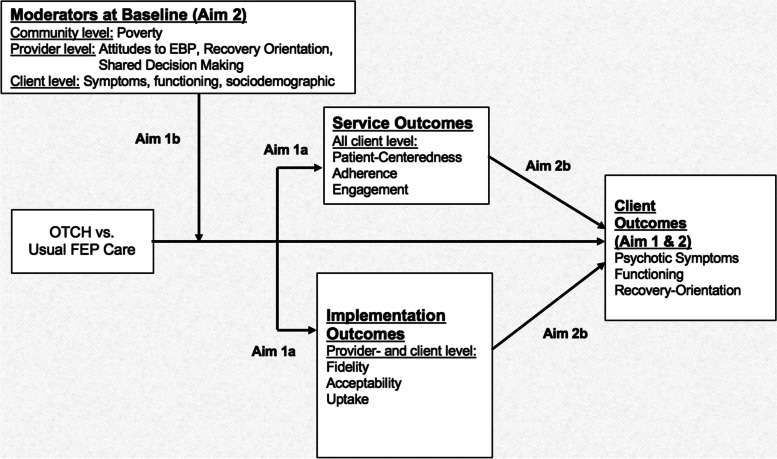
Conceptual model for analysis

Figure 3 presents the conceptual model that guides our analysis. The model posits, first and foremost, that by 12 months’ implementation and service outcomes will reflect what should be seen in participants if the implementation is properly done, based on previous studies [2, 19, 40]. It also indicates that factors at community (e.g., % poverty), provider (e.g., attitudes to evidence-based practices), and participant (e.g., socio-demographics) levels could moderate the effect of OTCH on implementation and service outcomes. The model also posits that the effect of OTCH vs FEP Usual Care on clinical outcomes (symptoms, functioning, and recovery orientation) will be evident at 24 months. It further proposes that 12-month implementation and service outcomes will mediate 24-month clinical outcomes.

We will carry out all analyses on an Intention-to-treat basis. All tests will be two-sided with critical value *α*=0.05. Prior to analysis, we will examine all variables for outliers and inconsistencies and examine their distributions. The distributions of all continuous variables will be checked for normality, and transformations will be employed to normalize distributions, if necessary, before applying specific parametric techniques. Missing data will be imputed, using multiple imputation procedures in standard statistical software (e.g., Stata or SAS).

#### Aim 1a and Aim 1b analyses

We have selected 3 measures of implementation (fidelity, acceptability, and uptake) and 3 measures of service outcomes (patient-centeredness, adherence, and retention) that represent the active ingredients of CSC for people with FEP. These six measures reflect care processes that will be fundamental to the implementation of the OTCH program [13]. Tests for treatment differences between the intervention arms on each of the six outcomes will be performed using a hierarchical linear model (HLM) for each outcome including clinic-level random effects to account for clustering of participants within clinics and participant-level random effects to account for repeated measures on the same user over time. The primary fixed effects will be an indicator for intervention status (OTCH, FEP Usual Care), time (12 or 24 months), and intervention*time. The magnitude and statistical significance of the beta coefficients from the HLMs will be used to test whether implementation and service outcomes differ between intervention arms at 12 and 24 months. If descriptive analyses identify any baseline differences between randomized groups of participants at baseline, we will consider adding the respective covariate to the model to adjust for this.

We will examine multilevel factors that may moderate the implementation of OTCH (see Table 1). (1) *Community level*: there is evidence that access to and engagement with mental health services is low among those who reside in poor communities [41]. (2) *Provider level*: we hypothesize that more positive attitudes toward evidence-based practices and recovery orientation will be associated with more successful implementation of programs which have these characteristics like OTCH [42]. (3) *Participant level*: we also hypothesize that socio-demographics and baseline clinical state will be predictors of implementation and service outcomes based on prior studies with clinical outcomes [17]. We will test this aim by including all three types of moderators into the HLM utilized for Aim 1.a. Specifically, because we aim to test whether these factors differentially affect the implementation of the OTCH compared to FEP usual care (i.e., an interaction with treatment), we will incorporate a random intervention effect into the HLM and allow the factors to predict the random intervention effect at both the clinic and participant level. The magnitude and statistical significance of the beta coefficients for these factors will provide the test of them as treatment effect moderators.

#### Aim 2a and 2b analyses

We hypothesize that the OTCH program will be effective and that, over time, user-level psychotic symptoms, functioning, and recovery orientation will improve. Tests for treatment differences between the intervention arms on each outcome (symptoms, functioning, and recovery orientation) will be performed using an HLM including an indicator for intervention status, time (baseline, 12, 24 months), and intervention*time. The magnitude and statistical significance of the beta coefficient for the interaction will provide an assessment of the extent to which the change in psychotic symptoms, functioning, and recovery orientation since baseline over time differs by intervention arm.

Based on prior research [43–45], we have hypothesized that service and implementation outcomes at 12 months will mediate the impact of OTCH on clinical outcomes at 24 months. To establish service and implementation outcomes at participant- and provider-level at 12 months as mediators of the OCTH effect on user-level clinical symptoms, functioning, and recovery orientation at 24 months, in addition to finding intervention effects (OCTH vs FEP usual care) as in Aim 1a and Aim 2a, we must also establish that service and implementation outcomes at 12 months are associated with better participant symptoms, functioning, and recovery orientation at 24 months. The mediation effect [46] is estimated by taking the expected value of the total effect of OTCH vs FEP Usual Care on symptoms, functioning, and recovery, and subtracting the direct effect when service and implementation outcomes are considered. This indirect (or mediation) effect of the implementation factors will be estimated and tested by fitting a Structural Equation Model using MPlus version 7.4 which our lead statistician has extensive expertise. We will estimate mediation models that consider one implementation or service mediator at a time, as well as including all indicators of implementation or service simultaneously to identify independent effects. The bootstrapping method (available in Mplus) will be used to obtain standard errors, confidence intervals, and the test for statistical significance [47]. Even in an RCT, the association between the mediator and the outcome can be confounded by other time-varying confounders and recent developments in mediation analysis suggest the need for sensitivity analyses to explore the potential impact of unmeasured confounders. We will perform these sensitivity analyses which provide a range of plausible results that may have been obtained under different assumptions about unmeasured confounders [48].

### Study management

This study will have several teams in charge of the varying components. There is an overarching study steering committee that oversees all decisions regarding study procedures, the implementation of the study protocol, and data collection. The steering committee meetings are attended by all senior investigators and lead members of working groups. Should any decisions be made by the study steering committee which require an amendment to the study protocol, a modification is submitted to the University of Chile Ethical Committee (the lead IRB of the study) and the New York State Psychiatric Institute IRB. The updated protocol will be available in the study regulatory binder, as well as any new letters of approval from the regulatory bodies. Changes will also be specifically noted in steering committee meetings and working group meetings and will be included as part of regular training for study personnel.

Additionally, a local, independent Data and Safety Monitoring Board (DSMB) was established at the initiation of the study, including three experts in the areas of data management and biostatistics, mental health services in Chile, and global health. The DSMB reviews the study as it progresses, ensuring that researchers and intervention team members understand the processes in place to protect the safety of study participants, and to verify whether the study protocol and procedures are being followed correctly. The DSMB has reviewed and approved the current study protocol and will review the data collection as it progresses, during its biannual meetings.

In addition to the Steering Committee and the DSMB, the NIMH provides an independent auditor who conducts a local site initiation review, as well as annual monitoring visits throughout the life of the study. The auditor checks numerous aspects of study management and data collection, including recruitment and informed consent practices, all paper and electronic research records, and reviews any protocol deviations and reporting of serious/adverse events. Any findings are resolved and documented.

Moreover, an adaptation team will be responsible for performing initial and ongoing qualitative interviews with stakeholders at various levels to inform adaptations to the OTCH model. A clinical team will be responsible for determining clinical adaptations to be adopted, training all the providers OTCH and examining the trend in the data collected to provide feedback to OTCH teams about their implementation. A research team, directed by a Data Center Manager, will be responsible for all quantitative and qualitative data collection.

### Access to data

The final trial dataset will be provided to the PIs of the study. The NIMH data archive will be used as the permanent repository for the final dataset, to be shared with qualified investigators for research via the terms and process established by the NIMH.

### Dissemination policy

Aside from publishing and presenting results of key findings in professional journals and meetings, the OTCH PIs and steering committee are committed to work with key stakeholders, partners, and interested members of the community to explore findings as they relate to current policy and practice. It is our hope that the experiences offered by this study will be informative as well as to the broader global mental health community.

We will use standard criteria for authorship proposed by well-known organizations, e.g., ICMJE guidelines (https://www.icmje.org/recommendations/browse/roles-and-responsibilities/defining-the-role-of-authors-and-contributors.html). However, this might be modified to ensure inclusion of people who contributed much work but are not proficient in writing in English. We will follow NIH requirements for data sharing. Full protocol will be published and code for statistical analyses will be available on request. Participant-level data might be available as long as it is anonymized and in which individuals cannot be identified.

## Discussion

The data collected in this study will help researchers evaluate the effectiveness and cost of evidence-based FEP treatment in outpatient clinics in Chile and identify potential facilitators and barriers that might influence outcomes and implementation. This is the first time that a CSC model will be adapted for universal public services in a large public health system of care in Latin America. The current governmental mandates for delivering care to individuals experiencing FEP provide the higher-level support necessary for a successful implementation. Furthermore, given that professionals are required to provide treatment to this population, there is a higher likelihood that they are receptive to a model and associated training to make their work more structured and effective with participants. CSC models are utilized internationally; however, there are very few mental health centers implementing it in South America. As such, in addition to understanding how to effectively adapt and implement these models to the Chilean reality, it is possible that this study will also help inform the field on strategies for effectively disseminating CSC across other LMIC in the area.

## Data Availability

Anonymized data from the study will be available from the authors after the trial has ended. The current trial does not involve collecting biological specimens for storage.
